# Visualizing cellulase adsorption and quantitatively determining cellulose accessibility with an updated fungal cellulose-binding module-based fluorescent probe protein

**DOI:** 10.1186/s13068-018-1105-0

**Published:** 2018-04-09

**Authors:** Tian Li, Nan Liu, Xianjin Ou, Xuebing Zhao, Feng Qi, Jianzhong Huang, Dehua Liu

**Affiliations:** 1 Key Laboratory for Industrial Biocatalysis, Ministry of Education of China, Institute of Applied Chemistry, Department of Chemical Engineering, Tsinghua University, Beijing, 100084 China; 2 Engineering Research Center of Industrial Microbiology of Ministry of Education, College of Life Sciences, Fujian Normal University, Fuzhou, 350117 Fujian China; 3 Institute of Biophysics, Chinese Academy of Sciences, Beijing, 100101 China

**Keywords:** Cellulose accessibility, Enzymatic hydrolysis, Adsorption, Fluorescent probe protein, Cellulose-binding module

## Abstract

**Background:**

Cellulose accessibility to cellulases (CAC) is a direct factor determining the enzymatic digestibility of lignocellulosic cellulose. Improving CAC by pretreatment is a prerequisite step for the efficient release of fermentable sugars from biomass cell wall. However, conventional methods to study the porosimetry of solid materials showed some limitations to be used for investigating CAC. In this work, an updated novel fusion protein comprising a fungal cellulose-binding module (CBM) from Cel7A cellobiohydrolase I (CBH I) of *Trichoderma reesei* QM6 and a di-green fluorescent protein (GFP_2_) was constructed for quantitative determination of CAC.

**Results:**

The obtained probe protein had similar molecular size (e.g., weight) with that of Cel7A and could give detectable signal for quantitative analysis. Several construction strategies were compared with regard to the site of His-tag and order of CBM and GFP_2_ modules in the protein sequence, in order to achieve good expression quantity and usability of the probe protein. His6–CBM–GFP_2_ has been identified as the best probe protein for investigating the effects of structural features of cellulosic substrates on cellulose accessibility. Substrate samples with different contents of xylan, lignin, and degree of substitution of cellulose –OH by formyl group were obtained, respectively, by mild H_2_SO_4_ pre-hydrolysis, NaClO_2_ selective delignification, and treatment of filter paper cellulose with concentrated formic acid. The determined CAC was in a wide range of 0.6–20.4 m^2^/g depending on the contents of hemicelluloses, lignin, and formyl group as well as cellulose degree of crystallization.

**Conclusions:**

The obtained fusion probe protein could be used as a versatile tool to quantitatively investigate the impacts of biomass structural features on CAC and hydrolyzability of cellulose substrates, as well as nonproductive adsorption of cellulase enzymes on lignin.

**Electronic supplementary material:**

The online version of this article (10.1186/s13068-018-1105-0) contains supplementary material, which is available to authorized users.

## Background

The biomass recalcitrance constructed by the hierarchical cell wall structure imposes a great limitation to bioconversion of biomass [1]. Improving the cellulose accessibility to cellulases (CAC) is a prerequisite step for efficient enzymatic saccharification of cell wall cellulose [2]. Therefore, lignocellulosic biomass must undergo pretreatment to increase CAC, by either chemical or physical methods or their combinations. Many researchers have corroborated that increasing the CAC is of great importance for efficient enzymatic hydrolysis of cellulose [2–7]. Increase in specific surface area (SSA) usually leads to improvement of CAC. However, SSA is not completely equivalent to CAC, because not all of the pores are accessible for cellulase enzymes [8]. In general, SSA is governed by substrate porosity and pore structure [9], and the pore structures of lignocellulosic biomass are hierarchical and dependent on the cell wall organs at different levels [10]. Transport phenomena suggest that pore size should be at least in the range of 50–100 nm to allow sufficient penetration of cellulase enzymes [11, 12]. Therefore, determination of CAC is important and necessary to study the fundamentals of biomass recalcitrance and guide the pretreatment process to maximize cellulose conversion.

Various methodologies commonly used to study the porosimetry of solid powder have been employed to determine the pore structures and specific surface area of lignocellulosic biomass substrates [11–13]. For example, the classical Brunauer–Emmett–Teller (BET) method with molecular nitrogen as a probe molecule based on multilayer adsorption as a function of relative pressure has been widely used to determine the SSA of solid catalyst [14]. However, N_2_ is much smaller than cellulase enzymes in molecular size, resulting in overestimation of the CAC. Drying is usually required prior to determination, but conventional drying may cause hornification of the fibers and collapse of porous structures [15, 16]. Some other methods suitable for determining the pore volume or cellulose accessibility of wet biomass substrates have also been developed, such as the solute exclusion [7, 17, 18] and modified Simons’ staining techniques [19, 20]. However, the solute exclusion technique showed some disadvantages such as being laborious, unspecific to cellulose, without determination of the external surface area, being not suitable for the determination of absolute pore size and volume distribution, and easily affected by pore shape and osmotic pressure [11]. Therefore, to more accurately determine CAC, we proposed that the used probe molecules at least must meet the following criteria: (1) having a similar molecular size to that of cellulase enzymes, or the key component of cellulases, such as cellobiohydrolase; (2) having good water solubility in aqueous system; (3) specifically recognizing cellulose; and (4) providing detectable signal for quantitative assay. The first criterion is the most important because the pores in lignocellulosic substrates are various in size and structure. Only the suitable probe molecules having similar molecular size to cellulases can well estimate CAC. Smaller probe molecules may cause overestimated accessibility, while oversized probes may have underestimated the accessibility (see Additional file 1: Fig. S1).

In the past few decades, the use of labeled cellulase or CBMs with GFP, red fluorescent protein [21], or FITC/TRITC [22–24] has been developed to probe and visualize the structure of cellulose and cell wall at microscopic level. More recently published papers can be found for the use of cellulases or polymers (such as dextrans) labeled with organic dyes to visualize the porosity of cellulosic substrates [25, 26], and the depolymerization of various cellulosic materials [27–29]. Nevertheless, only a few papers have been published on the quantitative determination of cellulose accessibility using fluorescence labeled CBMs [5]. However, the current CBM–GFP fusion protein used for determining CAC was constructed with bacteria CBM typically from *Clostridium thermocellum*. Nevertheless, the available commercial cellulases are usually produced by fungi, such as *Trichoderma reesei* (*Hypocrea jecorina*). The CBMs from bacteria and fungi are different in the structure as well as the recognized substrates. To date, several hundred CBMs have been identified and grouped into 45 families [21]. *C. thermocellum* CBM belongs to family 3 [30], while *T. reesei* CBM belongs to family 1 [31, 32]. Family 1 CBMs consist of around 35 amino acids that are highly conserved between the two types of cellobiohydrolases and the four types of endoglucanases of *T. reesei*, while family 3 CBMs are much larger than family 1 CBMs with 100–170 amino acid residues [33]. As found by Tomme et al. [34], CBMs from different families might occupy different numbers of 110 face cellobiose lattice residues of cellulose, ranging from 13 to 40. Lehtio et al. found that fungal family 1 CBMs as well as the family 3 CBM from *C. thermocellum* CipA have defined binding sites on two opposite corners of Valonia cellulose crystals (see the different faces of cellulose crystals of *I*_α_ allomorph in Additional file 1: Fig. S2) [30]. Therefore, to more accurately determine the accessibility of cellulose to fungal cellulases, it is better to develop a probe protein containing a fungal CBM and having similar molecular size to that of cellulase (e.g., CBH). The objective of this work is thus to design an updated probe protein comprising a fungal CBM from *T. reesei* QM6a for specifically recognizing cellulose and GFP(s) for visualized and quantitative analyses. The construction and expression strategies of the fusion protein were studied, and the obtained purified probe protein was further applied to quantitatively investigate the effects of biomass structural features on CAC.

## Results

### Verification of CBM-mediated specific adsorption of the fusion probe protein on cellulose

**Fig. 1:**
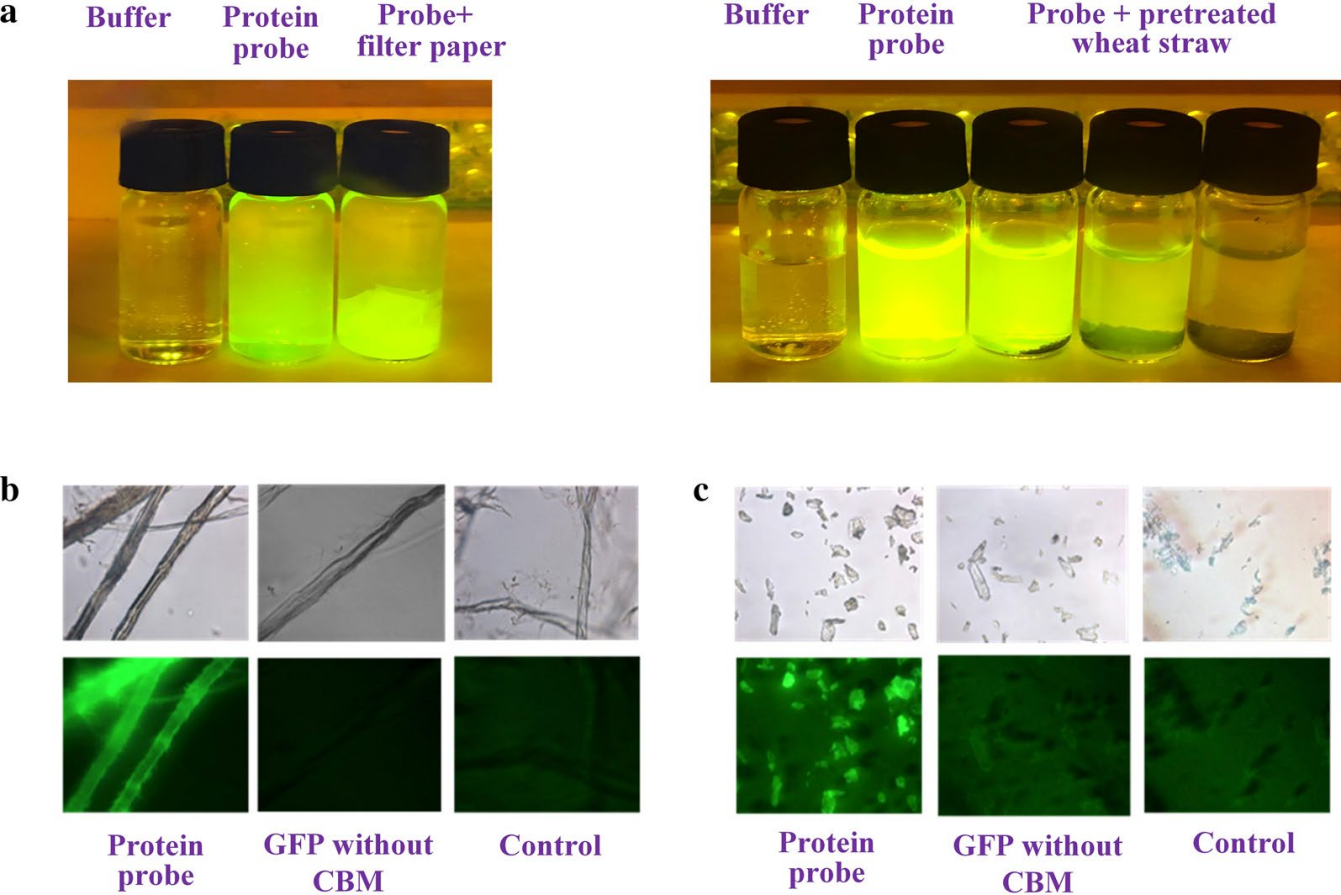
Adsorption of His6–GFP_2_–CBM fusion probe protein on filter paper (**a** left) and H_2_SO_4_-pretreated wheat straw (**a** right); and test of the specific adsorption of the probe protein on filter paper (**b**) and microcrystalline cellulose (**c**)

CBMs function as recognition modules to convey the catalytic modules of cellulases to the target cellulose substrate. Therefore, cellulase-mimetic probe for determination of CAC should have the function to specifically recognize cellulose. Solution containing His6–GFP_2_–CBM fusion protein showed strong green fluorescence as shown in Fig. 1a (left), in contrast to the buffer only control. After addition of filter paper, the fluorescent response of the suspension became weaker, while the filter paper gave strong green fluorescence, indicating that the probe protein could be adsorbed onto filter paper cellulose. The same phenomenon was observed for dilute H_2_SO_4_ pretreated wheat straw as shown in Fig. 1a (right), and lower fluorescence intensity was observed from the system with higher loading of pretreated wheat straw, indicating that more probe protein was adsorbed. To further verify the specific recognition of the probe protein, filter paper, and microcrystal cellulose (MCC) were used to adsorb fusion probe protein and di-GFP (GFP_2_) without CBM, respectively. As revealed by fluorescence microscopic images (Fig. 1b, c), obvious green fluorescence could be observed for probe protein, while no notable green fluorescence was observed for GFP_2_ protein without CBM and control (buffer solution). This result verifies that CBM is important to cellulose recognition, and the probe containing CBM could be specifically adsorbed on cellulose.

### Comparison of different construction and expression strategies to prepare the fusion probe protein

**Fig. 2:**
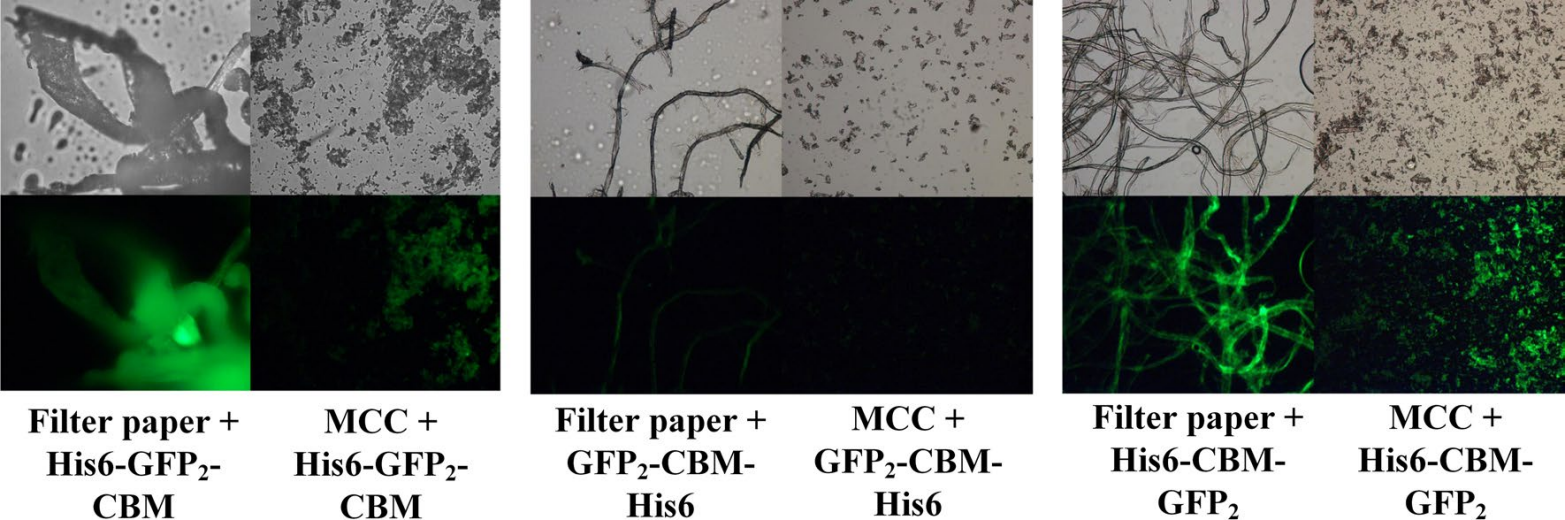
Adsorption of three fusion probe proteins His6–GFP_2_–CBM, GFP_2_–CBM–His6, and His6–CBM–GFP_2_ on filter paper and MCC. Images on the upper line was taken by optical microscope for comparison

Three fusion probe proteins, namely His6–GFP_2_–CBM, GFP_2_–CBM–His6 and His6–CBM–GFP_2_, were compared by specific adsorption on filter paper and MCC, as shown in Fig. 2. The results illustrated different surface adsorption behaviors of the three probe proteins with the same concentration of cellulose substrates. Strong green fluorescence response could be observed for His6–GFP_2_–CBM and His6–CBM–GFP_2_ proteins (Fig. 2, left and right, respectively) on both filter paper and MCC substrates. Nevertheless, very weak green fluorescence was observed for GFP_2_–CBM–His6 (Fig. 2, middle). The yields of the three protein constructs was found to be about 41.5, 1.1 and 57.2 mg/L culture broth, respectively, and the yield of GFP_2_–CBM–His6 was much lower. It was also found in the experiments that the  fusion protein His6–GFP_2_–CBM was prone to degrade to yield proteins or peptides with lower molecular weights. Fortunately, His6–CBM–GFP_2_ appeared to be stable, and thus it was selected as the best strategy for further analysis.

### Applications of the fusion probe protein to investigate the effects of biomass structural features on cellulose accessibility

CAC is greatly affected by the structural features of substrate. Particularly, the presence of hemicelluloses and lignin, and cellulose crystalline structure are the primary hindrance to CAC. Therefore, various pretreatments have been developed to improve CAC by removing hemicelluloses and/or lignin, or reducing cellulose crystallinity, with associated alteration of substrate physical structures. The novel fusion probe protein (His6–CBM–GFP_2_) was further employed to quantitatively investigate the effects of main structural features on CAC and enzymatic saccharification of cellulose, in combination with other characterizing approaches.

However, it should be noted that hemicelluloses and lignin are usually present in the pretreated substrates, which may also adsorb the probe protein thus interfering the adsorption of the probe molecules on cellulose. To determine whether hemicelluloses can significantly adsorb cellulases, we measured the adsorption of cellulase (Novozyme Cellic CTec2) on beechwood xylan (Sigma Aldrich) (see Additional file 1: Table S1). It was found that at low xylan concentration (2.5 g/L), the protein adsorption can be neglected since no reduced protein concentration in the liquid phase was detected. The percentage of protein reduction in the liquid phase became higher at low cellulase concentration or higher xylan concentration in the system. The amount of protein adsorbed on xylan was determined to be 2.2–4.3 mg/g xylan, which is much lower than that adsorbed by cellulose (16.3 mg/g cellulose). The probe protein was further used to confirm the adsorption of the probe molecules on xylan, as shown in Additional file 1: Fig. S3. No significant change in the fluorescence intensity was observed visually even as xylan concentration increased to 20 g/L. Determination of fluorescence intensity by fluorophotometer revealed that the signal intensity (diluted for 100 times) decreased from 27,291 ± 215 RFU of control (without addition of xylan) to 24,840 ± 168, 25,969 ± 315, and 24,995 ± 221 RFU, i.e., decreasing by 9.0, 4.8, and 8.4% for addition of 2, 10, and 20 g/L beechwood xylan, respectively. Moreover, in most cases, the xylan content in the pretreated substrates is low, and thus the adsorption of the constructed probe protein on xylan can be neglected. Nevertheless, the adsorption of cellulases on lignin is evident, and bovine serum albumin (BSA) can be used to block the nonproductive adsorption [5]. To confirm the efficiency of BSA blocking, wheat straw Klason lignin at concentrations of 2 and 6 g/L was employed to test the adsorption of the probe protein. As shown in Additional file 1: Fig. S4, BSA blocking indeed could reduce the adsorption of the probe protein on Klason lignin. At 2 g/L Klason lignin concentration, the fluorescence intensity decreased from 27,291 ± 215 RFU of control to 21,399 ± 188 RFU of the system with added Klason lignin but without BSA blocking. However, blocking with 5, 25, and 50 g/L BAS resulted in fluorescence intensities of 28,452 ± 267, 28,833 ± 321, and 28,889 ± 331 RFU, respectively. Nevertheless, at higher lignin concentration (6 g/L), the fluorescence intensities for the systems without BSA blocking and at 5, 25, and 50 g/L BAS blocking were 18,557 ± 121, 21,342 ± 182, 24,679 ± 278, and 27,822 ± 345 RFU, respectively, indicating that higher BSA concentration was needed for completely blocking the lignin nonproductive adsorption. Nevertheless, for the determination of CAC for samples with higher lignin content, for example, the dilute acid-pretreated sample, the solid concentration used was 2 g/L. Thus, 5 g/L BSA could efficiently block the nonproductive adsorption.

### Effects of hemicelluloses on cellulose accessibility

**Fig. 3:**
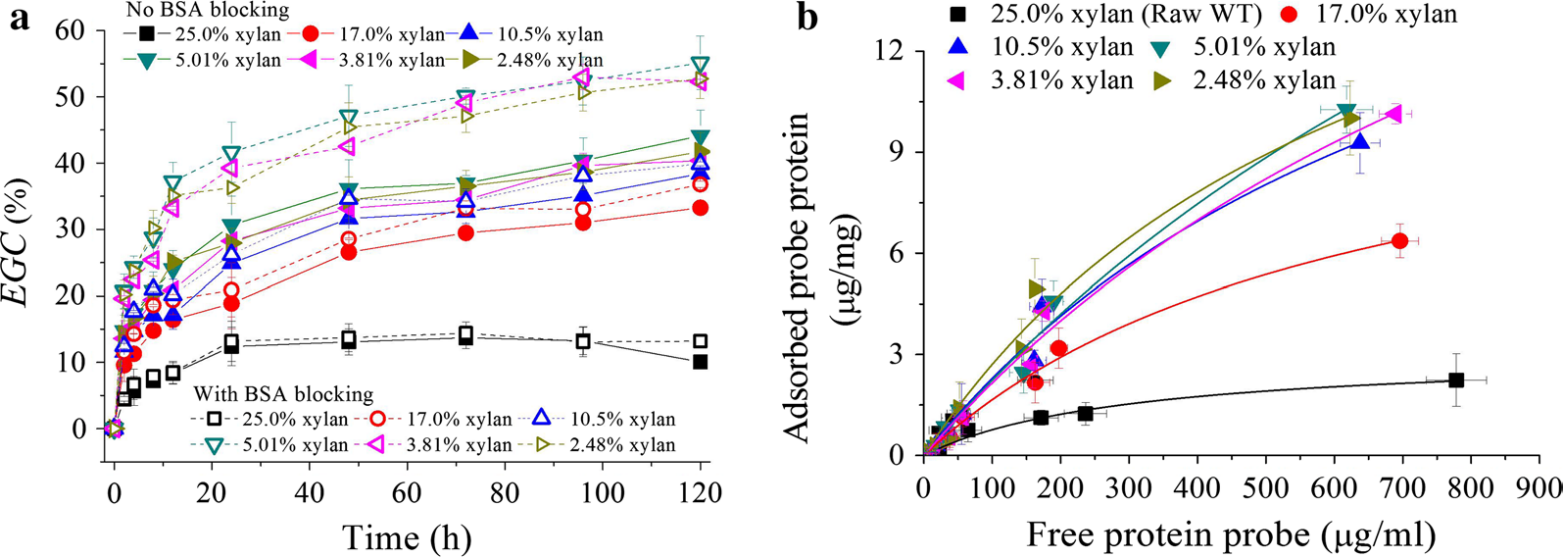
Effects of xylan removal by H_2_SO_4_ pretreatment on enzymatic glucan conversion with cellulase loading of 15 FPU/g solid (**a**); and Langmuir adsorption of probe protein on the BSA blocked substrates (**b**). BSA blocking was performed at 5 g/L concentration for Langmuir adsorption

Hemicelluloses (primarily present as xylan in grass biomass) can be removed by dilute acid hydrolysis with mineral acid such as H_2_SO_4_. However, to minimize the lignin melting and structure modification, H_2_SO_4_ pretreatment should be performed at low temperature [27]. Samples with different xylan contents were prepared under pretreatment using 0.4–8.0% H_2_SO_4_ at 120 °C. As shown in Table 1, by changing H_2_SO_4_ concentration, xylan content decreased, while glucan content increased, but the lignin content in the treated solid remained similar. Xylan removal was in the range of 30–95%, and the removal of total lignin (including acid soluble lignin and acid in-soluble lignin) was in the range of 5–30% depending on acid concentration used in the hydrolysis. The enzymatic hydrolysis of pretreated samples shown in Fig. 3a indicated that the enzymatic glucan conversion (*EGC*) increased generally with the decreasing xylan content. The raw wheat straw had a low *EGC* (< 13%) even after an extended incubation (120 h). However, for the 3% H_2_SO_4_ pretreatment, xylan content decreased to 5.0%, and corresponding *EGC*@120 h increased to 44.1%. Further decrease in xylan content showed no further positive effect on *EGC*, but, on the contrary, somewhat even reduced *EGC* as shown in Fig. 3a. This was probably because the obtained samples with different xylan contents under H_2_SO_4_ pretreatment still had high lignin contents, and nonproductive adsorption of cellulase enzymes on lignin could lead to decrease in *EGC*. Therefore, 5 g/L bovine serum albumin (BSA) was added to the hydrolysis system to block lignin’s nonproductive adsorption. As shown in Fig. 3a, increase in *EGC* indeed was observed with the addition of BSA; however, the degree of improvement was dependent on xylan content. For example, *EGC* could be enhanced by 5% at high xylan content (> 17%), while by 15% at low xylan content (< 5%).

**Table 1:** Selective removal of xylan by dilute sulfuric acid pretreatment of wheat straw under mild condition and the CAC determined by His6–CBM–GFP_2_ probe protein based on Langmuir adsorption

H_2_SO_4_ con. (wt%)	Contents of polymeric components			Fitted Langmuir equation parameters and CAC			
	*Gln* (%)	*Xln* (%)	*Lig* (%)	*A*_max_ (μg/mg)	*K*p	*R* ^2^	CAC (m^2^/g)
MCC	–	–	–	47.1 ± 19.2	0.00652 ± 0.00417	0.8721	9.3 ± 3.8
Raw wheat straw	35.1 ± 1.4	25.0 ± 0.8	27.9 ± 1.5	3.1 ± 0.6	0.00332 ± 0.00114	0.9547	0.6 ± 0.1
0.4	49.9 ± 0.2	17.0 ± 0.6	30.4 ± 0.4	12.2 ± 1.12	0.00157 ± 0.00032	0.9904	2.4 ± 0.2
1	55.2 ± 0.9	10.5 ± 0.2	31.3 ± 0.3	21.5 ± 2.9	0.00119 ± 0.00042	0.9806	4.3 ± 0.6
3	56.5 ± 1.1	5.01 ± 0.3	33.4 ± 0.3	33.8 ± 4.0	0.00071 ± 0.00031	0.9865	6.7 ± 0.8
5	62.9 ± 0.7	3.81 ± 0.2	32.5 ± 1.6	27.5 ± 3.5	0.00085 ± 0.00032	0.9851	5.5 ± 0.7

Samples were first treated by 5 g/L BSA to block the nonspecific adsorption of the probe protein on lignin

*Gln* glucan (cellulose) content, *Xyl* xylan content, *Lig* lignin content

Fluorescence microscopic images (Additional file 1: Fig. S5) showed that the probe protein was adsorbed on the surface of the pretreated substrates. The adsorption of probe protein could be described by Langmuir adsorption curves as shown in Fig. 3b. Corresponding fitted parameters are shown in Table 1. MCC was used for comparison. The maximal amount of protein adsorbed (*A*_max_) increased with the decreasing xylan content, and correspondingly, CAC increased. The raw wheat straw had a CAC of only 0.61 m^2^/g; however, xylan removal greatly increased CAC to 2.43–6.71 m^2^/g depending on the H_2_SO_4_ concentration used for pretreatment. The highest CAC of 6.71 m^2^/g was obtained by 3% H_2_SO_4_ pretreatment with 5% xylan content in the pretreated substrates. This result was in accordance with that observed by enzymatic hydrolysis (Fig. 3a). FTIR spectra analysis (Additional file 1: Fig. S6A) demonstrated that the acetyl groups that usually link as side chains in hemicellulose were removed as the hydrolysis of xylan by H_2_SO_4_ pretreatment, because the bands at 1740 cm^−1^ ascribed to C=O stretching became much weaker. XRD analysis (Additional file 1: Fig. S6B) illustrated that the polymorph of cellulose was not altered by H_2_SO_4_ pretreatment, while the crystallinity of the pretreated samples increased from 61.0% of raw wheat straw to 61.5–69% of treated substrates depending on H_2_SO_4_ concentration, mainly due to the removal of amorphous fraction such as xylan. Surface morphology characterization by SEM (Additional file 1: Fig. S6C) revealed that xylan removal by H_2_SO_4_ made the substrate more porous and coarse. Therefore, the results indicated that the increase in CAC by xylan removal via H_2_SO_4_ pre-hydrolysis pretreatment was primarily attributed to the increasing porosity of the substrates with the associated increase in specific surface area. However, as shown in Fig. 3a, the improvement of *EGC* was still limited (~ 45%) when no BSA blocking was used, suggesting that only a part of the cellulose was exposed, and the remaining part was still “blocked” by other components, particularly lignin. Chen et al. also reported similar conclusion [35].

### Effects of lignin on cellulose accessibility

**Fig. 4:**
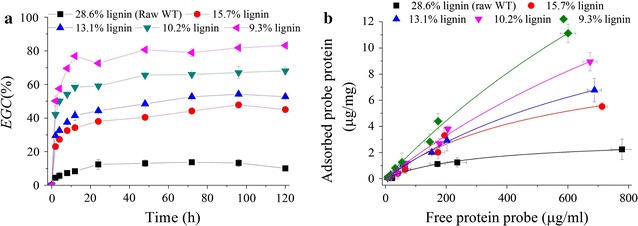
Effects of lignin removal by sodium chlorite pretreatment on enzymatic glucan conversion with cellulase loading of 15 FPU/g solid (**a**); and Langmuir adsorption of the probe protein (**b**). BSA blocking was performed at 5 g/L concentration for Langmuir adsorption

Lignin has been considered as an important cell wall component contributing to the biomass recalcitrance. Physically, it acts as a “glue” filling the remaining gaps of cell wall and excludes water from the polysaccharide environment [12]. Lignin can be selectively removed by sodium chlorite oxidative pretreatment under mild condition (75 °C). As shown in Table 2, by sodium chlorite pretreatment for 0.5–3 h, samples with different lignin contents were obtained. Corresponding glucan and xylan contents increased, and the loss of polysaccharides was less than 4%. Enzymatic hydrolysis results (Fig. 4a) demonstrated that the cellulose digestibility was greatly improved, and *EGC* increased with the decreasing lignin content. For example, *EGC*@12 h increased from 8.3% of raw wheat straw to 34.4, 41.5, 58.3, and 79.9% for samples with lignin contents of 15.7, 13.1, 10.2, and 9.3%, respectively. Clear green fluorescence was observed on the fiber surface of pretreated substrates with adsorbed probe protein after UV excitation (Additional file 1: Fig. S7). The estimated maximal amount of adsorbed probe protein on the pretreated substrates dramatically increased with the decreasing lignin content (Fig. 4b and Table 2).

**Table 2:** Selective removal of lignin by sodium chlorite oxidative pretreatment and the CAC determined by His6–CBM–GFP_2_ probe protein based on Langmuir adsorption

Sodium chlorite treating time (h)	Contents of polymeric components			Fitted Langmuir equation parameters and CAC			
	*Gln* (%)	*Xln* (%)	*Lig* (%)	*A*_max_ (μg/mg)	*K*_p_	*R* ^2^	CAC (m^2^/g)
Raw wheat straw	35.1 ± 1.4	25.0 ± 0.8	27.9 ± 1.5	3.1 ± 0.6	0.00332 ± 0.00114	0.9547	0.6 ± 0.1
0.5	50.7 ± 0.7	28.3 ± 0.2	15.7 ± 1.1	9.9 ± 1.9	0.00183 ± 0.00071	0.9589	2.0 ± 0.4
1	51.4 ± 0.5	29.6 ± 0.4	13.1 ± 0.3	16.4 ± 0.9	0.00102 ± 0.00015	0.9969	3.3 ± 0.2
2	53.1 ± 0.1	30.3 ± 0.1	10.2 ± 0.9	32.6 ± 2.9	0.00056 ± 0.00020	0.9923	6.4 ± 0.6
3	55.2 ± 1.0	31.6 ± 0.2	9.3 ± 2.9	45.8 ± 4.0	0.00054 ± 0.00021	0.9931	9.0 ± 0.8

*Gln* glucan (cellulose) content, *Xyl* xylan content, *Lig* lignin content

CAC increased by 2- to 14-fold depending on lignin content, ranging from 1.96 to 9.03 m^2^/g compared to 0.61 m^2^/g for the raw wheat straw. FTIR spectra (Additional file 1: Fig. S8A) showed that the band intensities at 1605, 1508, and 833 cm^−1^ which were attributed to the aromatic skeletal vibration of lignin became much weaker, corroborating the reduction of lignin content by sodium chlorite pretreatment. The intensity of the band at 1740 cm^−1^ assigned to unconjugated C=O bond stretching became stronger. This was probably because the aldehyde group of polysaccharides or hydroxyl group of lignin side chain was oxidized to form carboxyl group; or the benzene ring was oxidized to form quinone structure. However, this chemical structure modification did not show any negative effects on cellulose digestibility. XRD analysis (Additional file 1: Fig. S8B) illustrated that the polymorph of cellulose was not altered by sodium chlorite pretreatment, and the crystallinity of the pretreated samples was in the range of 57–67% depending on sodium chlorite pretreatment time, being similar to that of raw wheat straw. SEM imaging (Additional file 1: Fig. S8C) revealed that the substrate surface was greatly modified. The substrate surface became much coarse, and the cellulose fiber was even liberated at low lignin content.

Lignin also can nonproductively adsorb cellulases by hydrophobic, electrostatic and hydrogen bonding interactions [36, 37]. Several isolated lignins including sugarcane bagasse milled lignin (SBML), poplar alkaline lignin (PAL) and wheat straw Klason lignin (WSKL) which had different molecular weights and hydroxyl group contents (Additional file 1: Table S2) were added to filter paper hydrolysis system to study their inhibitive actions on cellulose conversion. All of these lignins showed notable inhibitive effects on cellulose hydrolysis with *EGC*@120 h decreased by 8–55% (Additional file 1: Fig. S9). Klason lignin showed the strongest inhibition followed by alkaline lignin, while milled lignin showed the lowliest negative effects. For example, when lignins were added at mount of 60% filter paper weight, the *EGC*@120 h were 23.6, 51.5 and 69.2% for Klason, alkaline and milled lignin added systems, respectively. Adsorption of fusion probe protein (Table 3 and Additional file 1: Fig. S10) corroborated that Klason lignin adsorbed the most probe protein, while milled lignin adsorbed the lowest. It was also found that the probe protein containing CBM was more prone to be adsorbed by lignin compared with the CFP_2_ (without CBM), particularly on alkaline and Klason lignins. The maximal amount of adsorbed protein increased by about four times for the probe protein containing CBM (Table 3).

**Table 3:** Determination of the maximal adsorption of GFP_2_ and probe proteins on several isolated lignins by Langmuir equation

Lignin	GFP_2_ (no CBM)			His6–CBM–GFP_2_		
	*A*_max_ (μg/mg)	*K*_p_	*R* ^2^	*A*_max_ (μg/mg)	*K*_p_	*R* ^2^
SBML	18.1 ± 3.3	0.0028 ± 0.0010	0.9907	19.0 ± 4.3	0.00516 ± 0.00165	0.8605
PAL	18.9 ± 4.9	0.1286 ± 0.0044	0.9146	105.7 ± 23.9	0.0456 ± 0.0120	0.9373
WSKL	34.3 ± 8.0	0.0421 ± 0.0124	0.8853	170.9 ± 20.0	0.01432 ± 0.0022	0.9793

No BSA blocking was used in these experiments

*SBML* sugarcane bagasse milled lignin, *PAL* poplar alkaline lignin, *WSKL* wheat straw Klason lignin

Therefore, the above results indicated that lignin removal greatly contributed to the exposure of cellulose surface thus significantly increasing the CAC with associated great increase in cellulose hydrolysability. The nonproductive adsorption of cellulases protein also could be reduced or eliminated through delignification and modification of lignin structure by sodium chlorite oxidative pretreatment.

### Effects of substitution of cellulose hydroxyl group by formyl group

**Fig. 5:**
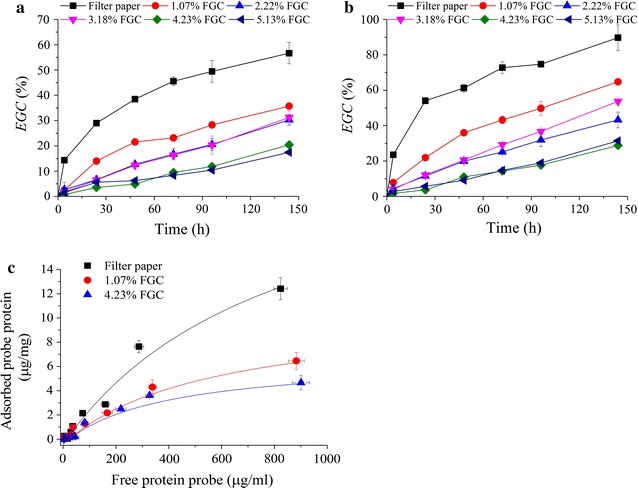
Effects of substitution of cellulose hydroxyl group by formyl group on enzymatic glucan conversion and green fluorescence response with adsorption of probe protein. **a** Enzymatic hydrolysis at cellulase loading of 5 FPU/g solid; **b** enzymatic hydrolysis at cellulase loading of 15 FPU/g solid; **c** Langmuir adsorption curves of probe protein on the substrates

The above results demonstrate that removing hemicelluloses or lignin can efficiently improve the CAC. Therefore, if hemicelluloses and lignin can be simultaneously removed in a one-pot process, the pretreated substrate would have greatly improved cellulose digestibility. Actually, our previous work demonstrated that Formiline pretreatment based on formic acid delignification and hydrolysis of hemicelluloses indeed could significantly enhance the cellulose hydrolysability of different lignocellulosic biomass [38–40]. However, formylation of cellulose took place during formic acid delignification, which might reduce the molecular recognition of cellulose substrates by cellulases [41]. The inhibitive action of formyl group introduced in formic acid treatment is the same as that of acetyl group present in hemicelluloses or introduced by Acetosolv pulping process, which has been considered as an important chemical factor limiting cellulose digestibility [42–44]. To investigate the impacts of substitution of cellulose hydroxyl group by formyl group on CAC, filter paper was treated by concentrated formic acid (70–88%) for different times (0.25–5 h) to obtain different formyl group contents. As shown in Fig. 5a, b, formyl group indeed significantly limited cellulose hydrolysis no matter whether high (15 FPU/g solid) or low (5 FPU/g solid) cellulase loading was used; however, the inhibitive effect became stronger at low cellulase loading. For example, at 15 FPU/g solid cellulase loading, *EGC*@144 h decreased from 89% for filter paper to 64.8% for substrate with 1.07% formyl group content, and 28.8% for sample with 4.23% formyl group content. Fluorescence micrographs (Additional file 1: Fig. S11) illustrated that the fluorescence response became much weaker at a high formyl group content (4.23%), suggesting a lower amount of probe protein adsorbed on the substrate. The determined values of CACs based on Langmuir adsorption curves (Fig. 5d) were 5.0 ± 0.6, 2.1 ± 0.2, and 1.36 ± 0.2 m^2^/g for samples with 0, 1.07, and 4.23% formyl group contents, respectively.

### Effects of cellulose crystallinity

Enzymatic hydrolysis of cellulose was also greatly affected by cellulose crystallinity. Typically, amorphous celluloses are 3–30 times faster to hydrolyze than high crystalline cellulose [45]. Our previous work has demonstrated that Formiline pretreatment could liberate fibers to obtain cellulose pulp. Posttreatment with cellulose-dissolving solvent further greatly improved cellulose digestibility [46]. The concentrated phosphorous acid (CPA) posttreated cellulose was very hydrolysable. Cellulose conversion reached 80% within 12 h of incubation with a low (7.5 FPU/g solid) cellulase loading (Additional file 1: Fig. S12). This great improvement of cellulose hydrolyzability was mainly ascribed to the decrystallization and depolymerization by CPA. The Formiline pretreated substrate had a crystallinity index of 57.7%, while CPA posttreatment reduced the crystallinity index to 23.6%. Strong green fluorescence was observed for both Formiline pretreated wheat straw and CPA posttreated substrates after adsorbing fusion probe protein (Additional file 1: Fig. S12D). CPA posttreated sample achieved a maximal amount of adsorbed probe protein of 103.1 μg/mg substrate, in contrast with 54.4 μg/mg substrate for Formiline pretreated substrate. Corresponding CACs were 10.8 and 20.4 m^2^/g for Formiline pretreated and CPA posttreated substrates, respectively. These data demonstrated that decrystallization was an efficient way to increase CAC for enzymatic hydrolysis.

**Fig. 6:**
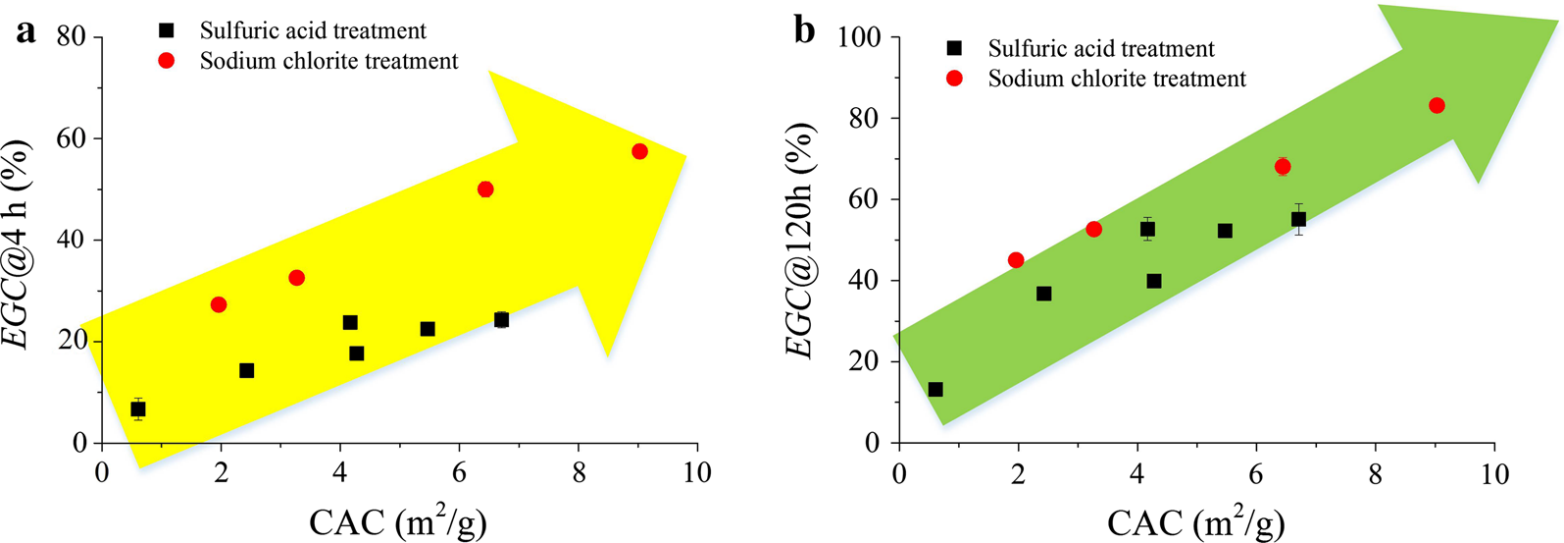
Relation between CAC and *EGC* for different samples obtained by H_2_SO_4_ and NaClO_2_ pretreatment of wheat straw to remove hemicelluloses and lignin of wheat straw, respectively. **a** Enzymatic hydrolysis for short time (4 h); and **b** enzymatic hydrolysis for long time (120 h). The *EGC* data for H_2_SO_4_ pretreated sample was based on BSA-blocked substrates

The relationships between CAC and *EGC* at 4 and 120 h were plotted for H_2_SO_4_- and NaClO_2_-treated substrates as shown in Fig. 6a, b, respectively. Significant positive correlation can be observed. The *EGC* at 4 h represented the initial hydrolysis rate of enzymatic hydrolysis, while *EGC* at 120 h represented the final conversion of cellulose. *EGC* generally increased with CAC linearly. However, the *EGC* may be different at the same CAC for different pretreatment processes. NaClO_2_ treatment obtained higher *EGC* than H_2_SO_4_ treatment, which indicated that under mild pretreatment condition, delignification probably was more efficient at improving cellulose digestibility.

## Discussion

CAC is the direct factor determining the cellulose hydrolysability. Increasing the CAC of lignocellulosic substrates is crucial to improve the enzymatic digestibility of cellulose. Generally, CAC can be enhanced by removing hemicelluloses and/or lignin, or decreasing biomass particle size and increasing the porosity, which also greatly increases the SSA of the substrates. Nevertheless, SSA is not equivalent to CAC, because not all of the pores in the biomass substrates are accessible to cellulase enzymes. Therefore, quantitative determination of CAC is important to study the fundamentals of biomass recalcitrance and guide the pretreatment process to maximize cellulose conversion. In the present work, we developed an updated GFP labeled Cel7A CBM that can be used to “mimic” the adsorption behavior of *T. reesei* cellobiohydrolase I (CBHI) on cellulose. The probe protein was designed to contain a CBM and two GFPs, and thus it could meet the “criterion” to use for quantitative determination of CAC because two GFPs (54 kD) of the probe had a similar molecular weight to the CBH catalytic module (52–55 kD) of *T. reesei *Cel7A. The experimental results demonstrated that the presence of CBM in the probe protein was crucial to a successful recognition and adsorption of the protein on cellulose. The adsorption of CBM on cellulose has been found to be thermodynamically spontaneous [47]. Therefore, the designed probe protein could be used under ambient condition without special processing. However, it should be noted that CBM can greatly increase the adsorption of CBH on cellulose surface, but the catalytic domain (CD) of CBH also contributes to the adsorption. Moreover, the probe protein may also show different surface charge or hydrophobicity, which could alter the behavior near the surface of cellulose. Nevertheless, compared with other currently reported methods to determine the porosimetry of lignocellulosic substrates, for example, BET method with N_2_ as the probe, mercury intrusion method with mercury as the probe, solute exclusion method with PEG or dextran as the probe and modified Simons’ staining method with direct blue and orange dyes as the probes, the probe protein developed in the present work at least has very similar molecular structure and weight to that of Cel7A.

However, it seems that the construction strategy of the gene sequence regarding to the site of His-tag, CBM and GFP_2_ from N-terminal to the C-terminal showed great influence on the stability of the protein after expression and purification. GFP_2_–CBM–His6 probe protein showed the weakest florescence response. This was primarily because the yield of this fusion protein was much lower (1.1 mg/L culture broth), which also can be directly corroborated by the fact that much weaker band was observed for this construct in the protein electrophoretogram (Additional file 1: Fig. S13) after the same protein recovery, concentration, and purification procedures. Therefore, the adsorbed probe protein on cellulose substrates was too little to give strong florescence signal. Moreover, the presence of N-terminal GFP domain might decrease the binding behavior of the proteins, since N-terminal tagging with GFP has been found to largely affect the whole fusion protein localization [48].

The constructed probe protein (His6–CBM–GFP_2_) could be used as a tool to visualize cellulase adsorption and quantitatively determine CAC of substrate samples with different structural features. Hemicelluloses and lignin are the major cell wall components contributing to the biomass recalcitrance and limiting cellulose accessibility. By selective removing hemicellulose with H_2_SO_4_ hydrolysis and removing lignin with sodium chlorite oxidation under mild condition, samples with different xylan and lignin contents were obtained. The determined CAC of these samples was 0.6–9.0 m^2^/g solid, which was in the reported SSA range (0.4–30 m^2^/g) of different pretreated substrates analyzed by different methods [11]. The CAC was well in accordance with the enzymatic glucan conversion. Both the initial enzymatic hydrolysis rate (*EGC*@4 h) and final cellulose conversion (*EGC*@120 h) increased as CAC with a linear relationship. However, the enzymatic hydrolysis of cellulose is greatly affected by the synergism of cellulase components and pore structure of the substrate with associated contents of hemicelluloses and lignin as well as pretreatment methods. For example, dilute acid pretreatment can create more pores by removing hemicelluloses, reducing particle size, and modifying cell wall structure, but not all of these pores are accessible for cellulases. The effects of these factors are usually very complicated with strong interactive effects, so the CAC cannot be expected to fit the *EGC* with a simple linear relationship. More research needs to be performed with regard to the analysis of substrate nanostructure and the impacts on CAC. However, the experimental results demonstrated that delignification seems to be more efficient for improving CAC under the mild pretreatment condition as demonstrated by the experimental results. This was because H_2_SO_4_ hydrolysis could achieve reduction of particle size, deformation of the cell shape, etching of the cell lumen surface, some fracture, and slight delamination of cell wall, with associated increase in porosity and specific surface area; however, most of the cell walls were still be “adhered” together due to the presence of the residual lignin which blocked a part of the cellulose. Nevertheless, sodium chlorite oxidative delignification resulted in significant etching, delamination, fracture, and even disappearance of the cell wall, thus greatly liberate cellulose from the packaging of hemicellulose-lignin complex [35]. Therefore, when delignification reached a certain degree, defibrillation could take place which greatly expose cellulose surface with significant increase in CAC.

In terms of the cellulose structure itself, the probe protein also showed good applicability to study the effects of cellulose –OH group on CAC. In the structure of cellulose molecule, there are three –OH groups at C2, C3, and C6 sites, corresponding to one primary (C6-OH) and two secondary groups (C2, C3-OH) in each glucose unit. However, substitution of the –OH group by formyl group greatly affected the cellulose digestibility. Glucan conversion decreased to only 28.8% when formyl group increased to 4.23% which corresponded to 8.7% substitution of total –OH group by formyl group. However, such a substitution had already demonstrated great decrease in *EGC*. Since the esterification rate of C6-OH is ten times faster than those of C2, C3-OH [12], it thus can be inferred that formylation of –OH groups of cellulose probably primarily took place at C6 site, and C6-OH might show the most important role on the CBM recognition toward cellulose. More details still need further investigation. The results also illustrated that substitution of –OH by formyl group could reduce the “apparent” CAC thus decreasing the adsorption of cellulase. However, this phenomenon might not be observed when using other probe molecules such as nitrogen, dextran or PEG to determine the CAC.

The probe protein also could be used to study the nonproductive adsorption of cellulase on lignin. By addition of isolated lignins to the pure cellulose hydrolysis system, it was found that Klason and alkaline lignins showed stronger inhibition to cellulose hydrolysis. This phenomenon can be explained by the fact that Klason lignin was a highly condensed lignin, while alkaline lignin had a higher phenolic hydroxyl group (Ph-OH) content (Additional file 1: Table S2); and condensed aromatic rings might enhance the hydrophobic interactions while Ph-OH could boost the hydrogen bonding [49]. It also indicated that the modification of lignin structure during pretreatment might make it more adsorptive to cellulase proteins by forming new functional groups such as Ph-OH and increasing lignin surface hydrophobicity via condensation reactions. The experimental results also demonstrated that the probe protein with CBM was easier to adsorb by lignin than GFP_2_ without CBM, which corroborated that CBM significantly contributed to the binding of cellulases to lignin matrix, particularly for cellobiohydrolase that is the major component of fungal cellulase complex. As revealed by Vermaas et al. [50], lignin preferentially binds to the specific residues (Y466, Y492, and Y493) on the CBM of *Tr*Cel7A. Those aromatic amino acids that are essential in CBM–cellulose interaction were also shown to contribute to lignin-binding via hydrophobic interaction [51]. However, it should be noted that lignin from different feedstocks and after pretreatment has very different abilities to adsorb protein. Thus, the adsorption of the probe protein to lignin needs to be checked if other feedstocks are investigated.

Therefore, the above discussion gives the conclusion that the developed novel probe protein can be applied as a versatile molecular tool to quantitatively investigate the effects of biomass structural features on cellulose digestibility, as well as the nonproductive adsorption of cellulases, especially CBM on lignin matrix. However, it should be noted that there are still some limitations for the developed probe protein to determine CAC. For example, the probe protein was only designed to mimic the adsorption behavior of CBH I of Cel7A; however, the degradation of cellulose needs synergetic actions of several cellulase components and the substrate structural features may affect the synergism of the enzymes. Moreover, the determination of the CAC with the probe protein was performed at low temperature (25 °C) and neutral pH (7.5); however, the enzymatic hydrolysis is usually performed at higher temperature (45–50 °C) and weak acidic condition (pH 4.8–5.5). The different temperatures and pH values may lead to some difference in the degree of the adsorption of the probe protein. Therefore, the probe protein still needs further modification to make it more mimetic to cellulases.

## Conclusions

An updated novel fusion probe protein comprising fungal CBM from cellobiohydrolase (Cel7A) of *T. reesei* QM6 and a di-green fluorescent protein (GFP_2_) were developed. The constructed protein was then employed to quantitatively investigate the impacts of biomass structural features on CAC. The determined values of CACs of various cellulosic substrates by the constructed probe protein were in a wide range of 0.6–20.4 m^2^/g depending on the contents of hemicelluloses and lignin, the degree of substitution of cellulose hydroxyl group by formyl group, as well as cellulose degree of crystallization. CAC could be well improved by removing xylan or lignin, while delignification seemed to be more efficient to expose cellulose. Substitution of –OH by formyl group reduced the adsorption of the probe protein by interference with the molecular recognition of CBM toward cellulose. Decrystallization greatly enhanced CAC. CBM played an important role to mediate the nonproductive adsorption of cellulase on lignin. The developed probe protein can effectively be used to determine the CAC of different substrates, but the relation with the *EGC* will still be highly determined by the properties of the substrate. However, the finding of this work can provide a new insight into the development of novel method to more accurately determine CAC. Nevertheless, the probe protein still needs further modification and update to better mimic cellulases such as the synergic action.

## Methods

### Cellulose substrate and chemicals

Filter paper was purchased from Hangzhou Special Paper Industry Co., Ltd (Hangzhou, China). Microcrystal cellulose (MCC) was provided by Sinopharm Chemical Reagent Co., Ltd (Beijing, China). Lignocellulosic biomass including wheat straw and sugarcane bagasse were collected from Shandong and Guangxi Provinces, China, respectively. The wheat straw had a glucan (cellulose) content of 35.1%, xylan content of 23.4% and lignin content of 27.9%. The sugarcane bagasse contained 38.1% glucan, 27.4% xylan, and 25.8% lignin. The cellulase enzymes, Cellic CTec2 was kindly provided by Novozymes (Beijing branch, China). The other chemicals used in the experiments were chemical-pure and purchased locally. The standard chemicals for HPLC were purchased from Sigma-Aldrich (Shanghai branch, China).

### Strains and culture conditions

*Escherichia coli* strains DH5a and BL21 (DE3) were used for gene cloning and expression of probe protein, respectively. The bacterial strains were cultivated in 2YT medium containing 1.6% (w/v) tryptone, 1% (w/v) yeast extract, and 0.5% (w/v) NaCl with supplementation of 50 mg/mL kanamycin sulfate when necessary in a rotary shaker at 37 °C and 220 rpm. *T. reesei* QM6a (ATCC 13631) was cultured on PDA medium. The strains used in this study are listed in Additional file 1: Table S3.

### Design of the fusion probe protein

A fragment containing two GFP (GFP_2_) genes was ligated to the CBM gene of *T. reesei* QM6a cellobiohydrolase I (CBH I) (Cel7A) by a linker that links CBM and cellulose catalytic module in *T. reesei* Cel7A (Additional file 1: Fig. S14). The GFP used in the experiment was stored in our lab with a molecular weight of 27 kD. Two GFPs (54 kD) were used to achieve a similar molecular weight to that of CBH catalytic module (52–55 kD) of *T. reesei* Cel7A. Therefore, by this design, the probe protein had several properties such as specific recognition of cellulose (by CBM), giving detectable signal (by GFP) and having similar molecular weight to that of CBH (by two GFPs).

### Construction of plasmids and preparation of the probe proteins

The detailed experimental information on construction of plasmids is provided in Additional file 1. In order to obtain good expression and usability of the fusion probe protein, three construction strategies were compared by changing the sites of His6, GFP_2_, and CBM in the protein sequence (from N to C terminals), which were termed His6–GFP_2_–CBM, GFP_2_–CBM–His6, and His6–CBM–GFP_2_, respectively. After expression in *E. coli*, the fusion proteins were isolated, recovered, and purified subsequently by ultrasonication, (NH_4_)_2_SO_4_ precipitation, and Ni–NTA column purification as per the detailed procedure provided in Additional file 1.

### Determination of CAC with fusion probe protein His6–CBM–GFP_2_

Determination of CAC with fusion probe protein His6–CBM–GFP_2_ was in accordance with the procedure described by Hong et al. [5] but with some modification. The adsorption was performed with 2–10 g/L cellulosic substrate loading at pH 7.4, 25 °C for 2 h. For substrates with high lignin contents, low substrate loading such as 2 g/L was used, while higher substrate loading was used for samples with low lignin loading. When the substrate contained lignin, it was firstly treated by 5 g/L BSA protein for 1 h to block the nonproductive and nonspecific adsorption of the probe by lignin. The amount of probe protein adsorbed was determined by measuring the change of fluorescent intensity of liquid phase before and after adsorption, and the protein concentration was then calculated using a standard curve correlating fluorescent intensity and protein concentration. The maximal amount of adsorbed probe protein on cellulosic substrate was determined by Langmuir equation:

1 $$Q_{\text{a}} = \frac{{A_{\text{max} } K_{\text{p}} Q_{\text{f}} }}{{1 + K_{\text{p}} Q_{\text{f}} }},$$

where *Q*_a_ is the amount of adsorbed probe protein (μg/mg); *Q*_f_ is the free protein concentration in the liquid phase (μg/mL); *A*_max_ is the maximal amount of probe protein that can be adsorbed by the substrate; and *K*_p_ is adsorption constant. In this work, the *A*_max_ was estimated by Eq. 1 with experimental data using software OriginPro 9.0. All tests at least in duplicate were performed at each point, and the average data were used for fitting. CAC thus can be determined by the following equation:

2 $${\text{CAC}} = \alpha A_{ \text{max} } N_{\text{A}} A_{\text{G2}}$$

where *α* is the number of cellobiose unit occupied by a CBM; *N*_A_ is Avogadro’s constant (6.023 × 10^23^ molecules/mol); and *A*_G2_ is the area of the cellobiose lattice on the 110 face (5.512 × 10^−19^ m^2^) [5]. It has been reported that a CBM of *T. reesei* CBH I occupies 38.7 cellobiose units when adsorbed on 110 surface [34]. Therefore, CAC can be calculated by Eq. 2 as long as *A*_max_ is determined.

### Enzymatic hydrolysis of the cellulosic substrate

The wet solid substrates were incubated at 50 °C, 150 rpm in 50 mM sodium acetate buffer (pH 4.8) in an air-bath shaker with a cellulase loading of 15 FPU/g solid. All experiments were performed at least in duplicate in 10 mL working volume at the initial solid consistency of 5% (g/100 mL). The enzymatic digestibility was characterized by enzymatic glucan conversion (*EGC*, %), defined as the percentage of glucan converted to glucose.

### Analytical methods

Determination of the main components, characterization of lignocellulosic biomass and pretreated substrates and the analysis of monosaccharides by HPLC were performed according to the methods described in previous work following NREL’s Laboratory Analytical Procedure [52]. Each determination was performed with tests in triplicate, and the data were reported as the mean values with standard errors.

The detailed methods for the spectroscopy (FTIR, XRD, and SEM) have been provided in Additional file 1. The fluorescence microscopic photograph was captured on a Nikon’s Eclipse E600 fluorescence microscope (Japan). The fluorescence intensity was determined using a HITACHI F-2500 fluorophotometer.

## Additional file

**Additional file 1.** Additional experimental information, tables and figures.

## Abbreviations

CBM: cellulose-binding module
CBH: cellobiohydrolase
CAC: cellulose accessiblity to cellulases
*DD*: degree of delignification
*EGC*: enzymatic glucan conversion
GFP: green fluorescent protein
GFP_2_: di-green fluorescent protein
MCC: microcrystalline cellulose
