# Supplementary material for: Visualizing cellulase adsorption and quantitatively determining cellulose accessibility with an updated fungal cellulose-binding module-based fluorescent probe protein
Source: Biotechnol Biofuels. 2018 Apr 9;11:105. doi: 10.1186/s13068-018-1105-0 (PMC5890345; doi:10.1186/s13068-018-1105-0)

**Additional information for**

**Visualizing cellulase adsorption and quantitatively determining cellulose accessibility by an updated fungal cellulose-binding module-based fluorescent probe protein**

Tian Li ^1, †^, Nan Liu^1,^ ^2, †^, Xianjin Ou ^3^, Xuebing Zhao ^1, *^, Feng Qi ^2, **^, Jianzhong Huang ^2^, Dehua Liu ^1^

^1^ Key Laboratory for Industrial Biocatalysis, Ministry of Education of China, Institute of Applied Chemistry, Department of Chemical Engineering, Tsinghua University, Beijing 100084, China.

^2^ Engineering Research Center of Industrial Microbiology of Ministry of Education, College of Life Sciences, Fujian Normal University, Fuzhou 350117, Fujian, China.

^3^ Institute of Biophysics, Chinese Academy of Sciences, Beijing 100101, China.

^*, **^ Correspondence and requests for materials should be addressed to X.Z. ([zhaoxb@mail.tsinghua.edu.cn](mailto:zhaoxb@mail.tsinghua.edu.cn)) or F.Q. ([f.qi@fjnu.edu.cn](mailto:f.qi@fjnu.edu.cn))

^†^ Both authors contributed equally to this work.

**Additional experimental information**

**Experimental procedure on construction of plasmids and preparation of the probe proteins**

Total RNA of *T. reesei* QM6a was isolated, purified and reverse transcribed to cDNA using RNA purification kit and cDNA reverse transcription kit (Omega Tech Inc., USA) according to the instructions described in a previous work. CBM-linker and GFP genes were amplified using the primers CBM-F, CBM-R and GFP_2_-F, GFP_2_-R, respectively. The fragment GFP_2_-CBM was obtained using overlap PCR with the primers GFP_2_-F, GFP_2_-R, GFP-CBM-F, GFP-CBM-R CBM-F and CBM-R listed in supplemental data Table S1. The fragment GFP_2_-CBM was digested using the restriction endonucleases EcoRI and HindIII, and ligated into pET28a, yielding the plasmid pET28a-HGC. The six-His tag was ligated to the N- or C-terminal of the resulting fusion protein. Then the fragment GFP_2_-CBM was amplified using the primers GFP_2_-F2 and CBM-R2, digested with NcoI and HindIII and ligated to pET28a for construction of the plasmid pET28a-GCH. The two pairs of primers CBM-GFP-F, CBM-GFP-R and GFP_2_-F3, CBM-R3 were used to obtain the fragment CBM-GFP_2_ by overlap PCR. The pET28a-HCG was constructed with ligation of the fragment CBM- GFP_2_ digested with EcoRI and HindIII. 100 μl of competent *E. coli* strains DH5a and BL21 (DE3) cells were freshly prepared by the CaCl_2_ method and mixed with 10 μl each of the constructed DNA ligation products, incubated on ice for 10min, and heat shocked at 42°C for 45 sec. The recombinant *E. coli* strains were then collected after incubation at 37°C for 12 h on the 2YT plate with 50 mg/mL of kanamycin sulfate. In order to obtain good expression and usability of the fusion probe protein, three construction strategies were compared by changing the places of His6, GFP_2_ and CBM in the protein sequence (from N to C terminals), which were termed as His6-GFP_2_-CBM, GFP_2_-CBM-His6 and His6-CBM-GFP_2_, respectively.

Each of the recombinant *E. coli* BL21 (DE3) strains cultured overnight was inoculated at 2% ratio into 20 ml 2YT medium (with 50 mg/mL of kanamycin sulfate) in a conical flask and cultured at 37°C in a shaker at 250 rpm. 0.2 mM isopropyl β-D-1-thiogalactopyranoside (IPTG, Merck, USA) was added until the culture reached a logarithmic phase (OD_600_=0.40~0.60). IPTG was added to a final concentration of 1 mmol/l and the culture temperature was adjusted down to 28°C. After 6 h induction, the cell pellets was collected after centrifugation at 8,000 rpm for 5 min, and re-suspended in 10 mL of PBS (140 mmol/l NaCl, 2.7 mmol/l KCl, 10 mmol/l Na_2_HPO_4_, 1.8 mmol/l KH_2_PO_4_) at pH 7.6. The cell suspension is lysed by sonicating on ice (Ultrasonic Fisher Sonic Dismembrator Model D100) at a 50% maximum strength for 5 min. The cell lysate was centrifuged at 12000g for 10 min and the supernatant was precipitated with addition of 50% saturated (NH_4_)_2_SO_4_. The released fusion proteins His6-GFP_2_-CBM, GFP_2_-CBM-His6 and His6-CBM-GFP_2_ were further purified using the Ni-NTA column (Merck, USA) with eluate containing 20~60 mM imidazole. The fusion protein solutions were dialyzed in a 50 mM sodium citric buffer (pH 6.0~6.5).

**Fourier transform infrared spectroscopy (FTIR)**

FTIR spectroscopic analysis was performed using a Thermo Scientific Nicolet iN10 FTIR Microscope (Thermo Nicolet Corporation, Madison, WI) equipped with a liquid nitrogen cooled MCT detector. Acetone-dried samples were embedded in KBr pellets with an approximate concentration of 1 mg/100 mg KBr. Scans were conducted at 400–4000 cm^-1^. Before data collection, background scanning was performed for correction.

**X-ray diffraction**

The crystallinity of the samples was determined by X-ray diffraction (XRD) using XRD-6000 instrument (Shimadzu, Japan). The X-ray diffractograms were recorded from diffraction angle (2*θ*) of 5° to 50° at a scanning speed of 5°/min with Ni-filtered Cu Kα radiation (χ= 1.54 Å) at 40 kV and 40 mA. The crystallinity index (*CrI*) was calculated using the following expression: *CrI*=(*I*_002_-*I*_am_)/*I*_002_×100%, where *I*_002_ is the intensity of peak at a 2*θ* angle close to 22.5° and *I*_am_ is the scattering intensity of amorphous fraction at a 2*θ* angle close to 18°.

**Scanning electron microscope**

Surface morphology analysis of the pretreated solids was performed using scanning electron microscope (SEM). A Hitachi S-3400N II (Hitachi, Japan) instrument operated at 15 kV was used. Prior to imaging, the samples were sputter-coated with a thin layer of gold to make the fibers conductive, avoiding degradation and buildup of charge on the specimen. Images were obtained at magnifications ranging from 45× to 30000× depending on the feature to be traced.

**Additional tables and figures**

**Additional tables**

**Table S1** Adsorption of cellulase (Novozyme Cellic CTec2) protein on beechwood xylan (Sigma Aldrich)

| Cellulase concentration (mg/L) | Xylan concentration (g/L) | Protein reduced in the liquid phase (%) | Protein adsorbed on xylan (mg/g) |
| --- | --- | --- | --- |
| 850 | 2.5 | ND | ND |
|  | 12.5 | 3.95±0.36 | 2.69±0.25 |
|  | 25 | 9.60±0.85 | 3.26±0.29 |
| 425 | 2.5 | ND | ND |
|  | 12.5 | 11.55±0.92 | 3.93±0.31 |
|  | 25 | 16.81±0.65 | 2.86±0.11 |
| 213 | 2.5 | ND | ND |
|  | 12.5 | 24.44±1.14 | 4.16±0.19 |
|  | 25 | 26.19±0.90 | 2.23±0.08 |

ND: no reduced protein concentration was detected

**Table S2** Molecular weights and hydroxyl group contents of several isolated lignins used in the experiments

| Lignin | $\bar{M_{w}}$ | $\bar{M_{n}}$ | $\bar{M_{w}}/\bar{M_{n}}$ | Total –OH (%) | Ph-OH (%) |
| --- | --- | --- | --- | --- | --- |
| SBML | 2610 | 1290 | 2.03 | 7.86 | 3.95 |
| PAL | 1510 | 1420 | 1.07 | 6.92 | 5.34 |
| WSKL | Highly condensed | Highly condensed | / | 5.96 | / |

SBML: sugarcane bagasse milled lignin; PAL: Poplar alkaline lignin; WSKL: Wheat straw Klason lignin; Ph-OH: Phenolic hydroxyl group content.

**Table S3** Strains, plasmids and primers used in this study

| **Strains** | **Genotype/description** | **Reference or source** |
| --- | --- | --- |
| *E.coli* DH5a | F^-^ λ^–^endA1 glnV44 thi-1 recA1 relA1 gyrA96 deoR nupG  Φ80dlacZΔM15 Δ(lacZYA-argF)U169, hsdR17(rK^-^ mK^+^) | QIAGEN |
| *E.coli* BL21 (DE3) | F^-^ ompT hsdS(rB^-^ mB^-^) dcm^+^ Tetr gal λ(DE3) endA Hte | QIAGEN |
| *E.coli* BL21-HGC | harboring pET28a-His6-2GFP-CBM | By this study |
| *E.coli* BL21-GCH | harboring pET28a-2GFP-CBM-His6 | By this study |
| *E.coli* BL21-HCG | harboring pET28a-His6-CBM-2GFP | By this study |
| *Trichoderma reesei* QM 6a | Genomic source of CBM | ATCC 13631 |
| **Plasmids** | | |
| pET28a | Expression plasmid, Kan^R^ | Novogen |
| pET28a-HGC | pET28a derivative expression plasmid with His6-2GFP-CBM | By this study |
| pET28a-GCH | pET28a derivative expression plasmid with 2GFP-CBM-His6 | By this study |
| pET28a-HCG | pET28a derivative expression plasmid with His6-CBM-2GFP | By this study |
| **Primers** | | |
| **Designation** | **Sequence (5’—3’)** |  |
| GFP_2_-F: | ATGCGTAAAGGCGAAGAGCT |  |
| GFP_2_-R: | TTCGCCTTTACGCATTTTGTACAGTTCATCCAT |  |
| GFP-CBM-F: | ACTGTACAAAATGCGTAAAGGCGAAGAG |  |
| GFP-CBM-R: | CTGCCGATGGGTCCTTTGTACAGTTCATCCAT |  |
| CBM-F: | AACTGTACAAAGGACCCATCGGCAG |  |
| CBM-R: | TTACAAGCACTGAGAGTA |  |
| GFP_2_-F2: | CATGCCATGGATGCGTAAAGGCGAAGAGCT |  |
| CBM-R2: | GACaagcttTTACAAGCACTGAGAGTAGTAAGG |  |
| CBM-GFP-F: | GACggatccGGACCCATCGGCAGCACTGGCA |  |
| CBM-GFP-R: | GCATgaattcCAAGCACTGAGAGTAGTAAGG |  |
| GFP_2_-F3: | TCAGTGCTTGGAATTCATGCGTAAAGGCGAAG |  |
| CBM-R3: | GACaagcttGGGTCCTTTGTACAGTTCATC |  |

**Additional figures**

**Fig. S1** Schematic diagram for determination of cellulose accessibility of lignocellulosic substrates with different probe molecules (Adapted from Beecher et al., In: Lucia LA and Rojas O (Eds), The Nanoscience and Technology of Renewable Biomaterials, 2009, Chapter 3, pp 61-90)

**Fig. S2** Schematic diagram of the organization of the cellulose chains and faces in the I_α_ allomorph of cellulose crystals (Lehtiö, J., Sugiyama, J., Gustavsson, M., Linder, M. y Teeri, T. T. (2003). The binding specificity and affinity determinants of family 1 and family 3 cellulose binding modules. Proceedings of the National Academy of Science of the United States of America 100, 484-489. Copyright © National Academy of Sciences)


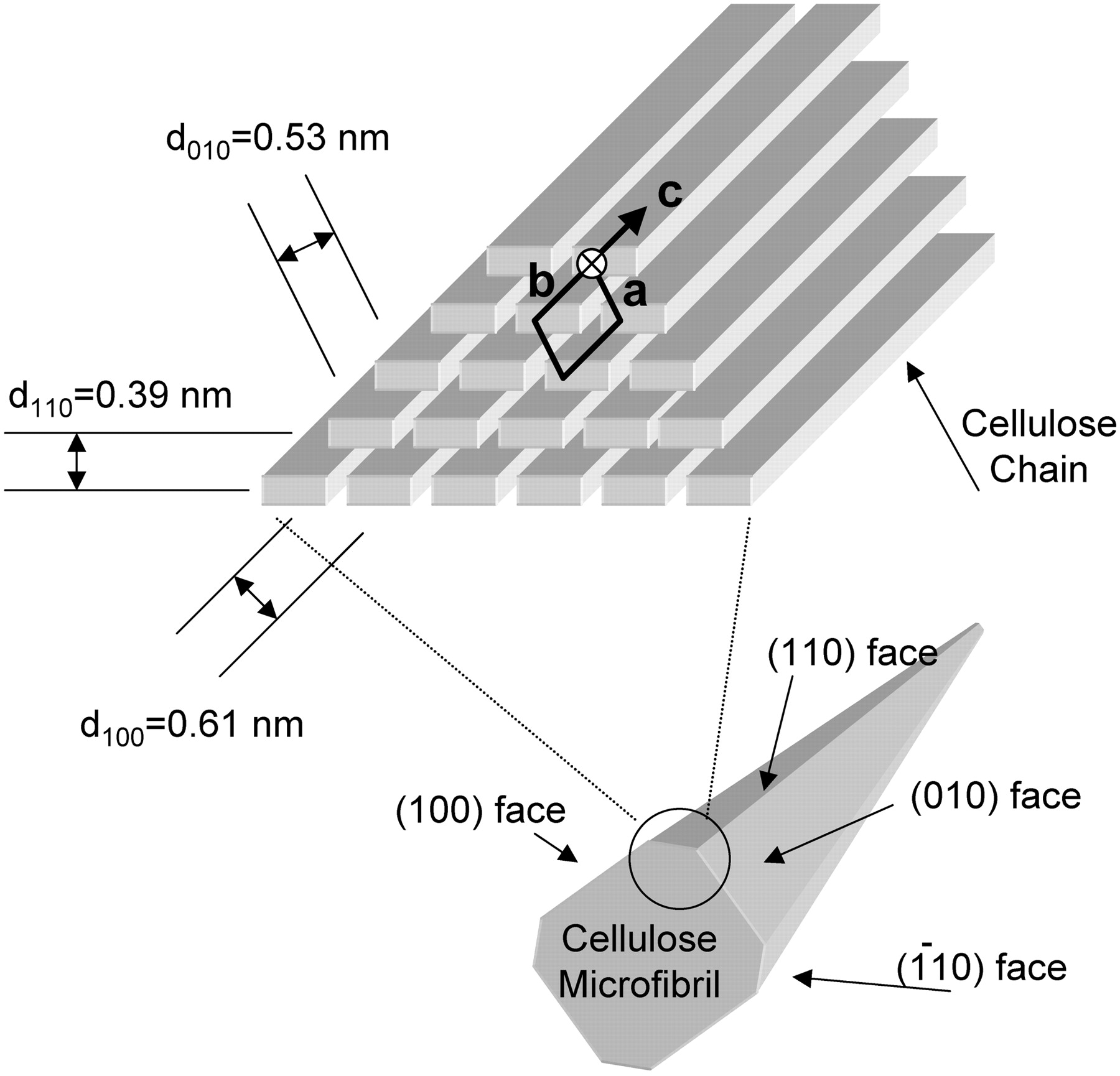


Fig. S3 Fluorescence images of probe protein solution with addition of isolated beechwood xylan at different concentrations. 1: no addition of xylan; 2: addition of 2g/L xylan; 3: addition of 10g/l xylan; and 4: addition of 20g/L xylan


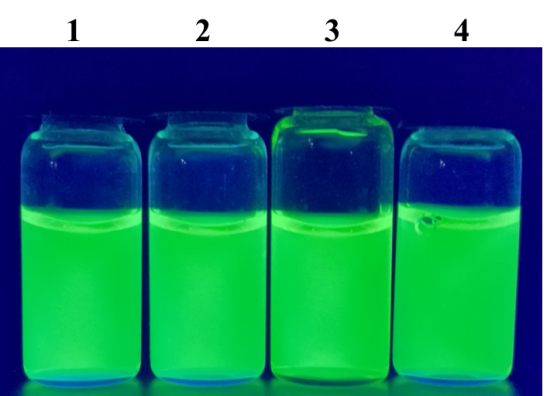


**Fig. S4** Effect of wheat straw Klason lignin and BSA blocking on the adsorption of probe protein. A: 2g/L Klason lignin concentration; B: 6 g/L Klason lignin concentration. 1: Control (probe protein solution with no Klason lignin); 2: no BSA blocking; 3: 5g/L BSA blocking; 4: 25 g/L BSA blocking; 5: 50g/L BSA blocking.

**
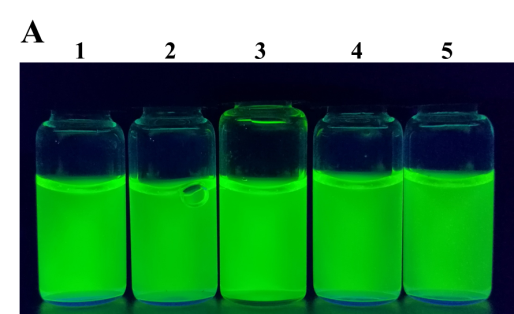
**

**
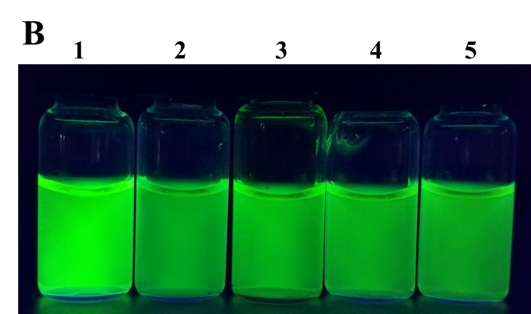
**

**Fig. S5** Green fluorescence response of the BSA blocked dilute H_2_SO_4_ pretreated wheat straw with different xylan content.

**Fig. S****6** FTIR spectra (A), XRD diagrams (B) and SEM images (C) of dilute H_2_SO_4_ pretreated wheat straw with different xylan content.

**Fig. S7** Green fluorescence response of the sodium chlorite pretreated wheat straw with different lignin contents.

**Fig. S8** FTIR spectra (A), XRD diagrams (B) and SEM images (C) of sodium chlorite pretreated wheat straw with different lignin contents.

**Fig. S9** Effects of several isolated lignins on enzymatic hydrolysis of filter paper. SBML: Sugarcane bagasse milled lignin; PAL: poplar alkaline lignin; and WSKL: Wheat straw Klason lignin.

**Fig. S10** Langmuir adsorption of GFP_2_ protein and probe protein (His6-CBM-GFP_2_) on different isolated lignins. SBML: Sugarcane bagasse milled lignin; PAL: poplar alkaline lignin; and WSKL: Wheat straw Klason lignin.

**Fig. S11** Green fluorescence response of filter paper cellulose and formylated cellulose

**Fig. S12** Effects of Formiline and concentrated phosphorous acid (CPA) pretreatments on enzymatic glucan conversion with different cellulose loading: (A)-Formiline pretreatment; (B)-Formiline+CPA pretreatment; (C)-Langmuir adsorption of protein probe; (D)-Green fluorescent micrographs; and (E)-substrate morphology by SEM images.


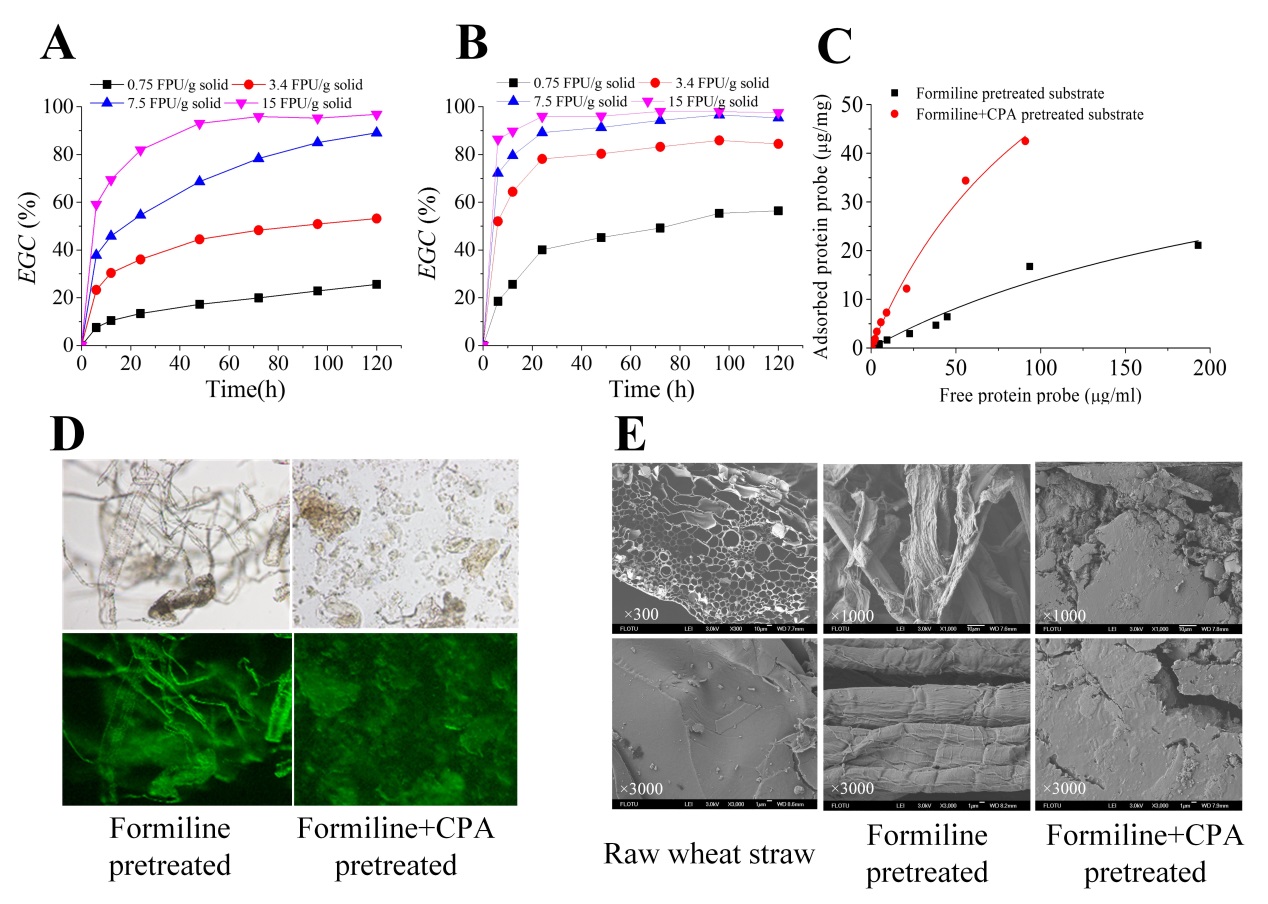


**Fig. S13** Electrophoretograms of the constructed fusion protein by SDS-PAGE. **(A)** For His6-GFP_2_-CBM: 0-Maker; 1-Fermentation broth without IPTG induction; 2-Fermentation broth with IPTG induction; 3-Resuspension of lysed cell precipitation; 4-Supernatant of cell lysate; 5-Purified protein diluted for 5 times; 6-Purified target protein diluted for 15 times; (B): For GFP_2_-CBM-His6: M-Marker; 1- Fermentation broth without IPTG induction; 2-Purified target protein; (C): For His6-CBM-GFP_2_: 0-Maker; 1-Fermentation broth without IPTG induction; 2-Purified target protein.

| **(A)**  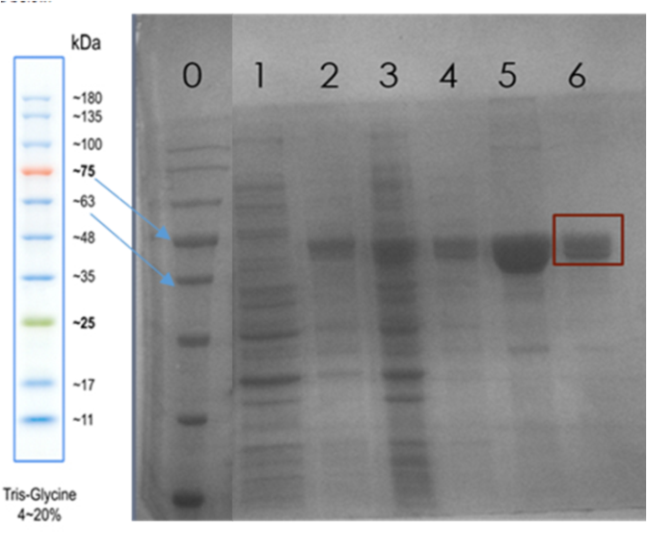 | **(B)**   |
| --- | --- |
| **(C)**   |  |

**Fig. S14** The design strategy for construction of the fusion probe protein.


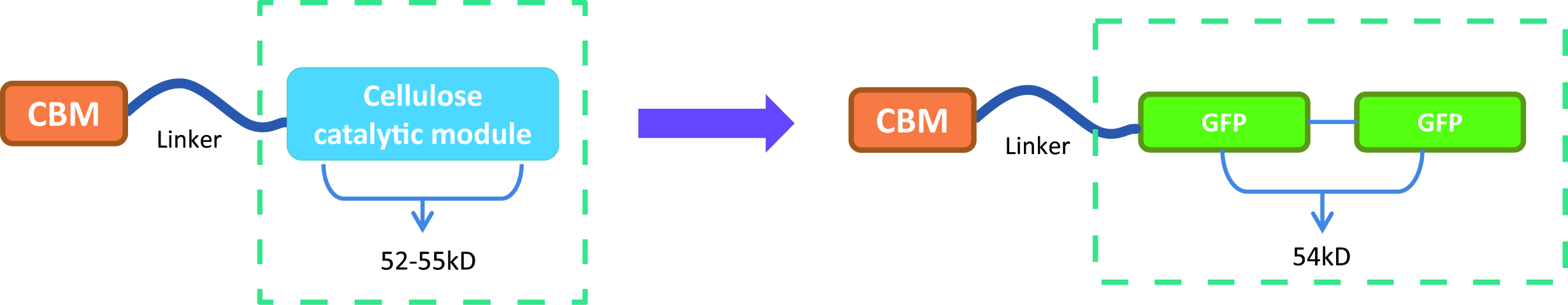

Supplement: Supplementary file 1 — Additional file 1. Additional experimental information, tables and figures. [file 13068_2018_1105_MOESM1_ESM.docx]
